# Platelet-membrane-coated nanoparticles enable safe and targeted thrombolysis with preserved neurovascular integrity

**DOI:** 10.3389/fphar.2026.1825954

**Published:** 2026-05-11

**Authors:** Ziying Feng, Ogunleye Femi Abiola, Guojun Huang, Tianfang Kang, Fan Xu, Ping Gong, Junlei Chang, Hong Pan, Yinzhong Ma

**Affiliations:** 1 State Key Laboratory of Biomedical Imaging Science and System, Guangdong-Hong Kong Joint Laboratory for Metabolic Medicine, Shenzhen Institutes of Advanced Technology, Chinese Academy of Sciences, Shenzhen, Guangdong, China; 2 Neuroscience Research Center, Stroke Center, Department of Neurology, The First Hospital of Jilin University, Changchun, Jilin, China; 3 University of Chinese Academy of Sciences, Beijing, China; 4 Guangdong Key Laboratory of Nanomedicine, CAS-HK Joint Lab of Biomaterials, Institute of Biomedicine and Biotechnology, Shenzhen Institutes of Advanced Technology, Chinese Academy of Sciences, Shenzhen, Guangdong, China; 5 Department of Cell and Molecular Biology, Karolinska Institutet, Stockholm, Sweden

**Keywords:** biomimetic nanoparticles, ischemic stroke, platelet membrane coating, recombinant tissue-type plasminogen activator (rtPA), thrombolytic therapy

## Abstract

Recombinant tissue-type plasminogen activator (rtPA) is the only FDA-approved thrombolytic agent for acute ischemic stroke, yet its clinical utility is constrained by a narrow therapeutic time window, rapid clearance, and hemorrhagic transformation risk. Here, we developed a biomimetic nanothrombolytic comprising rtPA-conjugated, platelet-membrane-coated PLGA nanoparticles loaded with perfluorohexane (PNP-rtPA) to improve thrombus specificity and vascular safety. PNP-rtPA preserved rtPA enzymatic activity, achieved efficient fibrin clot lysis *in vitro*, and markedly enhanced thrombolysis in an electrical-stimulation-induced carotid thrombosis model. Using a refined proximal photothrombotic middle cerebral artery (MCA) occlusion mouse model that generates stable large-vessel thrombosis, we show that PNP-rtPA restored cerebral blood flow more effectively than free rtPA, reduced infarct volume, improved neurological outcomes, and increased short-term survival. Pharmacokinetic and imaging analyses demonstrated prolonged circulation and enhanced accumulation of PNP-rtPA in the injured cerebrovascular bed. Importantly, PNP-rtPA mitigated blood–brain barrier (BBB) disruption, preserved tight junction and basement membrane integrity, and reduced hemorrhagic transformation and edema. Correspondingly, astrocytic swelling, microglial activation, and systemic cytokine release were attenuated, indicating improved neurovascular protection. Together, these results establish platelet-membrane cloaking and nanoscale rtPA delivery as an effective strategy to reconcile thrombolytic efficacy with vascular safety. PNP-rtPA represents a translatable biomimetic platform for achieving precise and safe thrombolysis in ischemic vascular disease.

## 1 Introduction

Ischemic stroke remains one of the leading causes of death and long-term disability worldwide, accounting for nearly 85% of all cerebrovascular events (Collaborators, 2021). Rapid restoration of cerebral blood flow is essential for minimizing irreversible neuronal loss and improving functional recovery. Recombinant tissue-type plasminogen activator (rtPA) is currently the only FDA-approved thrombolytic agent for acute ischemic stroke and has been the mainstay of pharmacological recanalization for more than 2 decades (Yogendrakumar et al., 2024). However, its clinical efficacy is severely constrained by a narrow therapeutic time window, a short plasma half-life (<5 min), and the high risk of intracerebral hemorrhage associated with systemic fibrinolysis (Tsivgoulis et al., 2023). These intrinsic limitations result in suboptimal reperfusion and poor outcomes, especially when administration is delayed beyond the recommended time frame.

To overcome the shortcomings of free rtPA, various drug-delivery strategies have been developed, including polymeric nanoparticles, liposomes, micelles, and PEGylated nanocarriers (Li et al., 2024; Xu et al., 2020). These platforms aim to improve pharmacokinetics, prolong circulation, and reduce systemic side effects. Despite these advances, most systems fail to achieve a satisfactory balance between thrombolytic efficacy and vascular safety. Many synthetic carriers lack precise thrombus targeting, leading to off-target fibrin degradation and bleeding complications. Moreover, some formulations compromise the enzymatic activity of rtPA during encapsulation or fail to release the payload efficiently at the occlusion site. Therefore, an ideal delivery system should not only preserve rtPA bioactivity and extend its circulation time but also ensure selective accumulation at thrombotic lesions while minimizing disruption of the vascular endothelium and the blood–brain barrier (BBB) (Alotaibi et al., 2021; Kastrup et al., 2008).

In the broader context of biomimetic nanocarrier design, various cell membrane-based systems, including erythrocyte- and stem cell-derived platforms, have been explored to improve circulation and immune evasion (Fang et al., 2018). Among these, platelet membrane-based systems are particularly well suited for thrombus targeting, as platelets intrinsically recognize vascular injury sites and fibrin-rich clots (Gardiner and Andrews, 2014; Chatterjee et al., 2015; Huang et al., 2019; Li et al., 2022). Building on this concept, we constructed a biomimetic platelet-membrane-coated PLGA nanoparticle loaded with rtPA (PNP-rtPA). The design integrates the biological intelligence of platelet membranes with the controlled-release and mechanical stability of polymeric nanocarriers. In addition, the perfluorohexane (PFH) core provides structural robustness and ultrasound responsiveness, allowing potential image-guided or externally controlled release in future translational applications (Hu et al., 2022).

**FIGURE 1:**
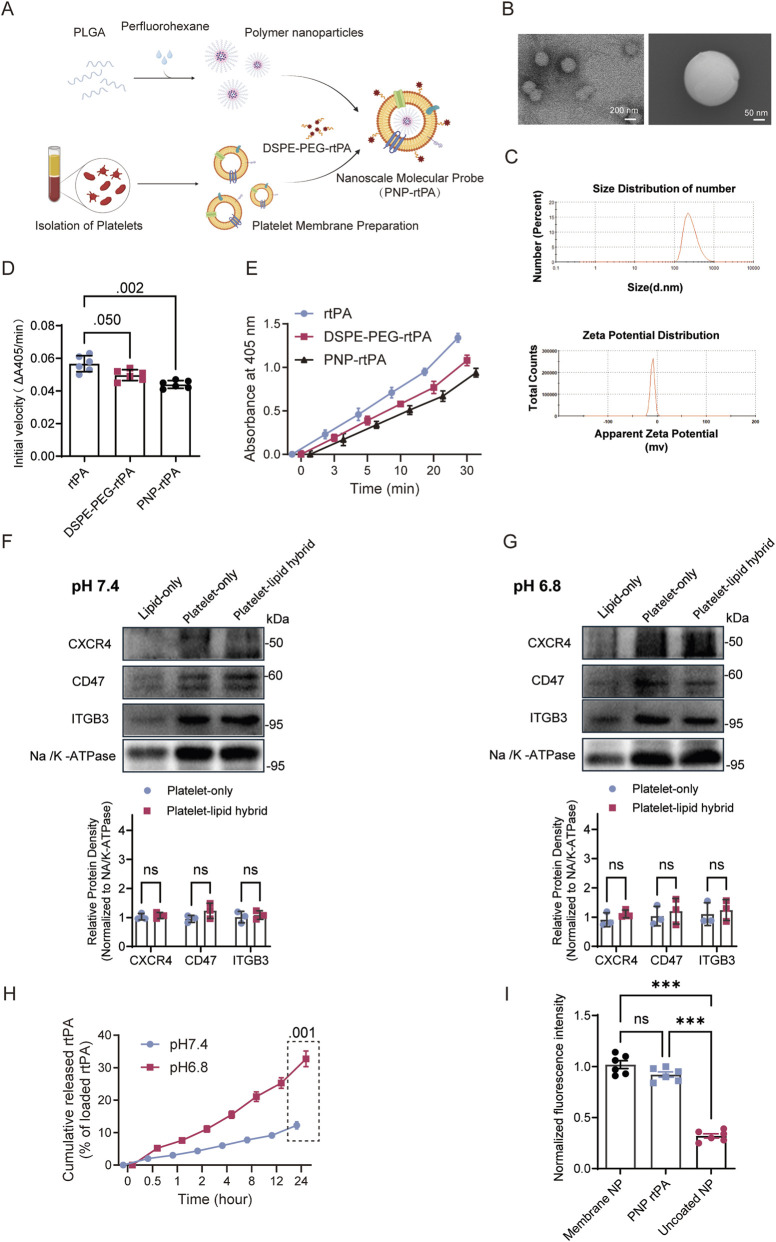
Fabrication and physicochemical characterization of PNP-rtPA thrombolytic nanoparticles. **(A)** Schematic illustration of the stepwise fabrication of recombinant tissue-type plasminogen activator (rtPA)–functionalized, platelet-membrane-coated poly lactic-co-glycolic acid (PLGA) nanoparticles. Perfluorohexane (PFH)-encapsulated PLGA nanoparticles were prepared, subsequently cloaked with isolated platelet membranes, and conjugated with DSPE-PEG-rtPA to obtain the final rtPA-PMC-PNP formulation. **(B)** Representative scanning-electron-microscopy (SEM) images showing the spherical morphology of rtPA-PMC-PNP at 200 nm and 50 nm scales. **(C)** Dynamic-light-scattering (DLS) analysis displaying the hydrodynamic-diameter distribution and ζ-potential profile of the nanoparticles. **(D)** Initial reaction velocity (ΔA405/min) of free rtPA, DSPE-PEG-rtPA, and PNP-rtPA determined from the linear phase of the chromogenic substrate assay. Data are presented as mean ± SEM (n = 6 independent replicates). **(E)** Time-dependent substrate cleavage kinetics measured as absorbance at 405 nm. Free rtPA, DSPE-PEG-rtPA, and PNP-rtPA were incubated with a chromogenic plasmin substrate, and absorbance was recorded at indicated time points (n = 6 independent replicates). **(F,G)** Western blot analysis of platelet-membrane proteins CXCR4, CD47, and ITGB3 in lipid-only, platelet-only, and platelet-lipid hybrid membranes under physiological (pH 7.4) and thrombus-mimicking (pH 6.8) conditions. Band intensities were normalized to Na^+^/K^+^-ATPase. Data are presented as mean ± S.E.M. (n = 6 biological replicates). **(H)** Time-dependent cumulative rtPA release/detachment from PNP-rtPA measured by ELISA under physiological (pH 7.4) and thrombus-mimicking (pH 6.8) conditions at 37 °C. **(I)** Quantification of clot-associated fluorescence intensity. DSPE-PEG-rtPA denotes rtPA conjugated to DSPE-PEG via amide coupling, whereas rtPA-PMC-PNP represents platelet membrane–coated, rtPA-conjugated, perfluorohexane-loaded PLGA nanoparticles. Data are presented as mean ± SEM (n = 6 independent replicates). Statistical analyses were performed using one-way ANOVA for single-time-point comparisons (panels D and I) and two-way repeated-measures ANOVA for time-course data (panels E and H), followed by appropriate post hoc tests. *P < 0.05, **P < 0.01.

Herein, we report the development and comprehensive evaluation of PNP-rtPA as a next-generation thrombolytic nanoplatform designed to reconcile efficacy and safety in ischemic stroke therapy. We hypothesized that the platelet membrane coating would confer thrombus affinity and immune evasion, while the polymeric core would protect rtPA from rapid degradation and enable sustained, localized fibrinolysis. Through a combination of *in vitro* assays and *in vivo* thrombotic models, we demonstrate that PNP-rtPA retains high enzymatic activity, achieves effective thrombus dissolution, and preserves BBB integrity and neurovascular homeostasis. This biomimetic approach not only enhances therapeutic performance but also provides a mechanistic framework for achieving “safe thrombolysis” — a long-standing goal in clinical stroke management. Our findings establish PNP-rtPA as a versatile and translatable nanotherapeutic platform for targeted vascular recanalization and neurovascular protection (Figure 1).

## 2 Materials and methods

### 2.1 Materials and reagents

Poly (D,L-lactide-co-glycolide) (PLGA, 50:50, MW 30–60 kDa) and 1,2-distearoyl-sn-glycero-3-phosphoethanolamine-N-[amino (polyethylene glycol)-2000] (DSPE-PEG-NH_2_) were purchased from Avanti Polar Lipids (United States). Perfluorohexane (PFH, 99%) was obtained from Sigma-Aldrich. Recombinant tissue-type plasminogen activator (rtPA, Actilyse®, Boehringer Ingelheim) was used as received. All other analytical-grade solvents and reagents were from Thermo Fisher Scientific unless otherwise specified.

Male C57BL/6J mice (8–10 weeks, 22–25 g) were obtained from the Experimental Animal Center of the Chinese Academy of Sciences. Mice were housed under standard SPF conditions with free access to food and water. Anesthesia and euthanasia were performed under deep isoflurane to minimize suffering. All animal studies were performed in accordance with the National Institutes of Health guidelines for use and care of live animals and were approved by the Institutional Animal Care and Use Committee of Shenzhen Institutes of Advanced Technology, Chinese Academy of Sciences (SIAT-IACUC-240326-YYS-MYZ-A2528). In addition, all animal experiments were conducted in strict accordance with the ARRIVE 2.0 guidelines to ensure rigorous and transparent reporting (Percie du Sert et al., 2020).

### 2.2 Physicochemical characterization

#### 2.2.1 Platelet isolation and PNP-rtPA preparation

Whole blood was collected from donor C57BL/6J mice by cardiac puncture into acid–citrate–dextrose (ACD-A; 1:9, v/v) to prevent coagulation. Platelet-rich plasma (PRP) was obtained by centrifugation at 200 *g* for 10 min at room temperature without brake. To minimize activation, PRP was supplemented with prostaglandin E1 (PGE1, 1 μM) and gently mixed. Platelets were pelleted at 800 g for 10 min, washed twice with calcium-free Tyrode’s buffer (134 mM NaCl, 12 mM NaHCO3, 2.9 mM KCl, 0.34 mM Na2HPO4, 5 mM HEPES, 5 mM glucose; pH 7.4), and resuspended at ∼1 × 10^9^ cells/mL.

For membrane preparation, platelet suspensions were subjected to hypotonic treatment (0.25× PBS, 30 min on ice) followed by three freeze–thaw cycles (−80 °C/37 °C) and brief probe sonication (3 × 5 s, ice bath). Membrane fragments were collected by centrifugation at 20 000 g for 30 min at 4 °C, washed with PBS, and extruded through 200-nm polycarbonate membranes (Mini-Extruder, Avanti) to obtain nanoscale vesicles. Total membrane protein was quantified by BCA assay (Thermo Fisher) and adjusted to the desired protein-to-core mass ratio for coating. Na^+^/K^+^-ATPase was used as a membrane-associated loading control for normalization, and verifying key membrane markers (CXCR4, CD47, and ITGB3) on the final platelet-membrane–coated nanoparticles by Western blot as described below. Membrane vesicles were stored at 4 °C and used within 7 days.

PLGA–PFH cores were synthesized by a modified double-emulsion solvent-evaporation method as previously described (Hernandez-Giottonini et al., 2020). Briefly, 100 mg PLGA was dissolved in dichloromethane containing 50 μL PFH, and the emulsion was sonicated (Scientz-IID, 20 kHz, 30 s). The primary emulsion was then dispersed into 2 mL of 1% PVA solution and re-sonicated to form a W/O/W emulsion, followed by stirring at room temperature for solvent evaporation. Nanoparticles were collected by centrifugation (12 000 g, 10 min) and washed with PBS.

Clinical-grade recombinant human rtPA (alteplase, Boehringer Ingelheim) was used for nanoparticle conjugation and as the free rtPA control. rtPA was covalently conjugated to DSPE-PEG-NH_2_ using EDC/NHS coupling chemistry (Hu et al., 2015). The resulting DSPE-PEG-rtPA was inserted onto nanoparticle surfaces through lipid adsorption. Platelet membranes were obtained from whole blood of donor mice by sequential centrifugation (800 g for 10 min, then 20 000 g for 30 min) and hypotonic lysis in 0.25× PBS. Membrane fragments were extruded (Avanti Mini-Extruder, 200 nm polycarbonate membranes) with rtPA-loaded nanoparticles at a 1:1 (w/w) protein-to-core ratio to yield PNP-rtPA. Samples were stored at 4 °C and used within 7 days.

rtPA loading efficiency was determined by ELISA (EH3900, Finetest) as the ratio of recovered rtPA to the initial input. For the high-input nanoparticle batches used for *in vivo* studies (target input 0.60 mg rtPA per tube), the recovered rtPA amount was 0.56 mg, corresponding to a loading efficiency of approximately 93%. Each batch was resuspended in 100 μl sterile PBS immediately before injection. Unless otherwise specified (pharmacokinetic experiment), all *in vivo* treatments were performed at an rtPA dose of 9 mg/kg (equivalent to 0.18 mg rtPA for a 20-g mouse). For PNP-rtPA groups, the injection volume was also 100 μl, corresponding to a final rtPA concentration of 2 mg/ml in the suspension, based on ELISA-quantified rtPA content after loading.

To assess batch-to-batch reproducibility of PNP-rtPA preparation, three independent nanoparticle batches were prepared using the same formulation protocol and target rtPA input. For each batch, rtPA loading efficiency was quantified by ELISA as described above. Batch variability was expressed as mean ± SD of loading efficiency across independent preparations. For selected batches, enzymatic activity was further evaluated using the chromogenic substrate assay, and the relative activity was normalized to free rtPA.

#### 2.2.2 DLS and SEM

The hydrodynamic diameter, polydispersity index (PDI), and zeta potential were measured using dynamic light scattering (DLS, Zetasizer Nano ZS, Malvern Instruments). Morphology was visualized using scanning electron microscopy (SEM, EGA 3 SBH, TESCAN CZ). Platelet-membrane proteins (CXCR4, CD47, and ITGB3) were verified by SDS-PAGE and Western blot (Bio-Rad Mini-Protean Tetra System) (Wang S. et al., 2020).

#### 2.2.3 Western blot analysis

Protein samples were boiled at 95 °C for 5 min, separated by SDS–PAGE on 6%–12% polyacrylamide gels, and electrophoretically transferred onto PVDF membranes (Millipore, United States). Membranes were blocked for 1 h at room temperature in 5% non-fat milk containing 0.05% Tween-20 in Tris-buffered saline (TBS-T), then incubated overnight at 4 °C with the following primary antibodies: Na^+^/K^+^-ATPase Rabbit pAb (1:1500, Cat. #A24883, Abclonal); CXCR4 Rabbit mAb (1:1200, Cat. #A19035, Abclonal); ITGB3 Rabbit pAb (1:900, Cat. #A24844, Abclonal); CD47 Rabbit mAb (1:1200, Cat. #A21904, Abclonal). After washing with TBS-T, membranes were incubated for 1 h at room temperature with HRP-conjugated goat anti-rabbit IgG (1:5000, Cat. #7074, Cell Signaling Technology). Immunoreactive bands were visualized using enhanced chemiluminescence reagents (ECL Plus, Thermo Fisher Scientific) and captured on a ChemiDoc MP Imaging System (Bio-Rad). Band densitometry was analyzed using ImageJ 1.52, and the intensity of each target protein was normalized to Na^+^/K^+^-ATPase. Each experimental group included 6 independent biological replicates. Representative blots were selected from one sample per group for figure presentation, whereas quantitative analysis was performed on all replicates.

#### 2.2.4 Enzymatic activity assay of rtPA

The enzymatic activity of rtPA after chemical conjugation and nanoparticle formulation was evaluated using a chromogenic substrate assay. Briefly, free rtPA, DSPE-PEG-rtPA, and PNP-rtPA were prepared at equivalent rtPA concentrations (1 μg/mL) in assay buffer (50 mM Tris-HCl, 100 mM NaCl, pH 7.4). A chromogenic plasmin substrate (S-2251, Chromogenix) was added according to the manufacturer’s instructions. The reaction mixture was incubated at 37 °C, and the absorbance at 405 nm was measured at defined time intervals using a microplate reader (Thermo Fisher Scientific). The initial reaction velocity (ΔA405/min) was calculated from the linear portion of the absorbance–time curve and used as an indicator of enzymatic activity. Relative activity was normalized to that of free rtPA. All measurements were performed with 6 independent replicates.

#### 2.2.5 *In vitro* rtPA release/detachment assay

PNP-rtPA was suspended in PBS adjusted to either pH 7.4 or pH 6.8 and incubated at 37 °C under gentle shaking to evaluate rtPA release/detachment under physiological and thrombus-mimicking conditions. The nanoparticle suspension was placed in low-protein-binding microcentrifuge tubes at an equivalent rtPA concentration of 2 mg/ml. At predetermined time points (0, 0.5, 1, 2, 4, 8, 12, and 24 h), samples were centrifuged at 12,000 g for 4 min to separate nanoparticles from the supernatant. The supernatant was collected and replaced with an equal volume of fresh prewarmed buffer to maintain sink conditions.The amount of released or detached rtPA in the collected supernatant was quantified using a human rtPA ELISA kit (EH3900, Finetest) according to the manufacturer’s instructions. Cumulative rtPA release/detachment was calculated as the percentage of total loaded rtPA detected in the supernatant over time. All experiments were performed using at least three independent nanoparticle preparations, with 6 replicates per condition.

#### 2.2.6 *In vitro* clot binding assay

An *in vitro* clot binding assay was performed to evaluate whether PEG-rtPA modification affects the thrombus-targeting capability of platelet-membrane-coated nanoparticles. Briefly, fibrin-rich blood clots were prepared by incubating freshly collected mouse platelet-poor plasma with thrombin (1 U/mL) and CaCl_2_ (10 mM) at 37 °C for 30 min in 96-well plates. Nanoparticle formulations were fluorescently labeled with FITC and adjusted to equivalent fluorescence intensity before use. Three groups were tested: (1) platelet-membrane-coated nanoparticles without PEG-rtPA modification (Membrane NP), (2) PNP-rtPA, and (3) uncoated nanoparticles without platelet membrane (Uncoated NP). Equal volumes of each formulation were added to preformed clots and incubated at 37 °C for 30 min under gentle shaking. After incubation, clots were washed three times with PBS to remove unbound nanoparticles. Clot-associated fluorescence was then measured using a microplate reader (Omega) with excitation/emission settings appropriate for FITC. Binding was quantified as relative fluorescence intensity normalized to clot area or to the Membrane NP group. All experiments were performed with at least 6 independent replicates.

### 2.3 *In vitro* thrombolytic activity

*In vitro* thrombolytic efficiency was assessed following established fibrinolysis protocols (Guan and Dou, 2021). Platelet-poor plasma (PPP) was prepared by centrifugation (800 g, 10 min) from fresh murine blood. Coagulation was induced with 10 mM CaCl_2_ and 1 U/mL thrombin in 96-well plates and incubated at 37 °C for 30 min. PNP-rtPA or free rtPA (equivalent to 1 μg rtPA) was then added to each well, and absorbance at 405 nm was recorded every 2 min for 60 min. The increase in optical density corresponded to the progression of fibrin degradation. The fibrinolytic rate was expressed as ΔA/Δt relative to the control.

### 2.4 *In vivo* thrombolytic efficacy in a common carotid artery (CCA) thrombosis model

The CCA thrombosis model was established as previously described with modifications (He et al., 2023). Mice were anesthetized with 2% isoflurane in O_2_, and the right CCA was exposed through a midline cervical incision. A platinum electrode connected to a thrombus formation tester (YLS-14B, Jinan Yiyan Science & Technology Development Co., Ltd) delivered a low-frequency direct current (0.5 mA, 1.5 min) to induce thrombosis. Mice were randomly assigned to receive vehicle, free rtPA (9 mg/kg, *i.v.*), or PNP-rtPA (equivalent rtPA dose).

Real-time blood flow was monitored using laser-speckle contrast imaging (RFLSI ZW, RWD Life Science). Perfusion intensity was quantified using RFLSI Analysis software as the ratio of post-treatment to baseline flow. After 1 h, arteries were harvested for histopathological evaluation (H&E, Leica DM6 B).

### 2.5 Therapeutic efficacy in a modified photothrombotic ischemic-stroke model

A modified proximal photothrombotic stroke model was established in male C57BL/6J mice to induce focal large-vessel thrombosis (Sun et al., 2020). Following intraperitoneal injection of Rose Bengal (50 mg/kg), a focused 532-nm laser beam (48 mW, 3 min, 0.7 mm diameter) was applied to the M3 segment of the middle cerebral artery through the intact skull in the left hemisphere. This short-duration, high-intensity illumination at the proximal MCA produced a stable thrombus and a reproducible occlusion pattern mimicking clinical large-vessel stroke pathophysiology. Stroke induction was confirmed using laser Doppler flowmetry; a decrease in ipsilateral cerebral blood flow to ≤60% of the contralateral side was required for inclusion. Animals failing to meet this criterion or experiencing surgical complications were excluded before randomization.

After 1 h of ischemia, mice received vehicle, free rtPA, or PNP-rtPA intravenously to mimic clinical thrombolytic therapy. In some animals, reperfusion after prolonged ischemia was performed to induce BBB disruption and hemorrhagic transformation, providing a model closely resembling clinical post-thrombolytic complications. Mouse Neurological Severity Score (mNSS, 0–14 scale) were assessed by blinded investigators at 48 h post modeling (Li et al., 2000).

#### 2.5.1 TTC staining

At 48 h, brains were harvested and sectioned (2 mm thickness), and incubated in a 2% solution of 2,3,5-triphenyltetrazolium chloride (TTC) (Sigma-Aldrich, MO, United States) at 37 °C for 20 min. The stroke areas were then quantified using ImageJ software (ImageJ, NIH, MD, United States). To correct for cerebral swelling (edema) and prevent overestimation of the infarction volume, the following formula was used (McBride et al., 2015):

Corrected infarction volume = Contralateral hemisphere volume - (ipsilateral hemisphere volume - infarction volume).

#### 2.5.2 Determination of BBB integrity

BBB permeability was evaluated using Evans Blue dye extravasation. Briefly, 100 µl of 2% Evans blue (cat. #E2129, Sigma-Aldrich, United States) was injected intravenously at 4 h prior to transcardial perfusion with 50 ml of ice-cold PBS to remove intravascular dye. Mice were then deeply anesthetized and perfused transcardially with 50 mL of ice-cold PBS to completely remove intravascular dye. Brains were separated into ipsilateral (ischemic) and contralateral (non-ischemic) hemispheres, homogenized in 1 mL of 50% trichloroacetic acid, and centrifuged at 10,000 rpm for 20 min at 4 °C. The resulting supernatant was diluted fourfold with absolute ethanol, and fluorescence intensity was measured using a microplate reader (Varioskan LUX, Thermo Fisher Scientific) at 620 nm excitation and 680 nm emission. Evans Blue content was calculated against a standard curve and expressed as nanograms of dye per milligram of brain tissue.

Hemorrhagic transformation was assessed by determining the hemoglobin levels in brain tissue using a Colourimetric assay kit (cat. #K219–200, BioVision Inc, United States). Mice were euthanized under deep anesthesia and transcardially perfused with 25 ml of ice-cold phosphate-buffered saline (PBS). The ischaemic hemisphere brain tissue was homogenized in distilled water using a FastPrep-24 5G homogenizer (MP Biomedicals, United States) for 1 min, followed by centrifugation at 13,400 g for 15 min. The supernatant was then mixed with the hemoglobin detector, and the absorbance was read at 575 nm using a spectrophotometer. Hemoglobin concentration was calculated from a standard curve generated using known concentrations of hemoglobin.

#### 2.5.3 Immunofluorescence analysis

Frozen coronal brain sections (10–12 μm) were mounted on Superfrost Plus slides (Thermo Fisher Scientific, United States) and air-dried at room temperature before rehydration in PBS. Sections were blocked in 5% normal goat serum containing 1% BSA and 0.5% Triton X-100 for 30 min, then incubated overnight at 4 °C with the following primary antibodies diluted in PBS containing 1% BSA and 0.3% Triton X-100: anti-mouse CD31 (1:600, Cat. #MAB1398Z, Merck Millipore), rabbit anti-Claudin-5 (1:100, Cat. #34–1600, Thermo Fisher Scientific), rabbit anti-ZO-1 (1:500, Cat. #21773-1-AP, Proteintech), rabbit anti-Laminin (1:100, Cat. #L9393, Sigma-Aldrich), rabbit anti-Collagen IV (1:200, Cat. #ab6586, Abcam), and other antibodies described in the respective figures. After washing with PBS containing 0.1% Triton X-100, sections were incubated for 1 h at room temperature with FITC- or Cy3-conjugated secondary antibodies (1:400, Jackson ImmunoResearch). Nuclei were counterstained with DAPI (Cat. #8961S, Cell Signaling Technology) and mounted with antifade reagent.

Fluorescent images were captured on an OLYMPUS CKX53 inverted microscope using identical exposure settings across groups. Signal area or integrated optical density (IOD) was quantified with Image-Pro Plus software (Media Cybernetics) and normalized to vascular area defined by CD31 immunoreactivity.

#### 2.5.4 Evaluation of neuroinflammation and safety

Astrocytic reactivity was assessed by immunofluorescence staining for glial fibrillary acidic protein (GFAP; 1:100, Cat. #12389, Cell Signaling Technology) and aquaporin-4 (AQP4; 1:200, Cat. #59678, Cell Signaling Technology). Microglial activation was evaluated using goat anti-Iba1 antibody (1:400, Cat. #ab289874, Abcam). The staining procedures were performed as described above for BBB immunofluorescence, including blocking, incubation, and fluorescence imaging under identical conditions. Quantitative analysis of integrated optical density (IOD) was conducted using Image-Pro Plus software, and data were normalized to the total field area within the peri-ischemic cortex.

Morphometric analysis of Iba-1–positive microglia was performed using ImageJ (NIH). Concentric circles were drawn at 2-μm intervals from the soma, and the number of dendritic intersections at each radius was quantified. The area under the intersection–radius curve (AUC) was defined as the Sholl index, representing overall dendritic complexity. Microglial density was determined by counting Iba-1–positive cells within defined cortical regions and normalizing to the total number of DAPI-positive nuclei in the same field to correct for variations in tissue thickness or cell packing. Data from 3–5 fields per section and three sections per animal were averaged to represent individual biological replicates.

Blood samples were collected from mice via cardiac puncture under deep anesthesia at the designated time points. The collected blood was allowed to clot at room temperature for 30 min and then centrifuged at 3,000 × g for 10 min at 4 °C to obtain serum. The supernatant (serum) was carefully collected and stored at −80 °C until further analysis. Serum levels of rtPA were quantified using commercial ELISA kits according to the manufacturer’s instructions. Cytokine levels (IL-1β, TNF-α, IFN-γ) were quantified in serum using multiplex ELISA kits: IL-1β (QT-EM0109, Finetest); TNF-α (EM0183, Finetest); IFN-γ (EM0093-HS, Finetest). Intracerebral hemorrhage was evaluated by hemoglobin assay (P0381S, Beyotime) and expressed as μg hemoglobin per mg brain tissue.

### 2.6 MRI acquisition and intensity kinetics analysis

Longitudinal *in vivo* magnetic resonance imaging (MRI) was performed to characterize the temporal evolution of ischemic lesions after photothrombotic middle cerebral artery occlusion (pMCAO). Imaging was conducted using a 9.4 T small-animal MRI system (uMR 9.4 T, United Imaging Healthcare). T_2_-weighted images were acquired using a spin-echo sequence with the following parameters: repetition time = 3000 ms, echo time = 33 ms, slice thickness = 0.8 mm, field of view = 20 × 20 mm, and matrix size = 256 × 256. Mice were anesthetized with 1.5%–2% isoflurane in oxygen and maintained at 37 °C using a thermostatically controlled animal bed equipped with respiratory monitoring. MRI scans were performed at 0.5, 1, 3, 6, 9, 12, and 24 h after occlusion to monitor lesion progression.

Signal intensity values were quantified using ImageJ software. Regions of interest (ROIs) were manually delineated on coronal sections to define the ischemic core, peri-infarct penumbra, and contralateral homologous region. The mean signal intensity within each ROI was measured, and relative signal changes were calculated by normalizing ipsilateral values to those of the contralateral hemisphere. The temporal evolution of mean T_2_-weighted signal intensity in each region was plotted to generate intensity–time kinetics curves, reflecting progressive tissue alterations from cytotoxic edema to infarct maturation. All analyses were performed by two independent investigators blinded to treatment allocation.

### 2.7 *In vivo* fluorescence imaging and biodistribution analysis

To visualize the pharmacokinetics and tissue distribution of the nanoparticles, we prepared two FITC-labeled formulations: (1) FITC-labeled PNP-rtPA, and (2) FITC-labeled tag-only control, which contained DSPE-PEG-FITC inserted into lipid nanoparticles but lacked both platelet-membrane coating and rtPA. For the tag-only control, DSPE-PEG-FITC (MW 10000; MCE) was directly inserted into the surface of control lipid nanoparticle through lipid adsorption to generate FITC-tagged nanoparticles without biomimetic coating. For FITC-labeled PNP-rtPA, the PLGA–PFH core nanoparticles were coated with platelet membranes and conjugated with rtPA following the procedure described above.

For small-animal imaging, C57BL/6J mice were intravenously injected with FITC-labeled PNP-rtPA or FITC-labeled PNP (equivalent to 0.5 mg/kg of FITC). Whole-body fluorescence imaging was performed at 0, 10, 30, and 60 min post-injection using an *in vivo* imaging system (Caliper ivis spectrum, Revvity) with excitation/emission filters set at 490/520 nm. Fluorescence intensity was quantified in regions of interest (ROIs) encompassing major organs using Living Imaging software.

### 2.8 Statistical analysis

All data were presented as mean ± SEM. Statistical analyses were performed using GraphPad Prism (version 10.0, GraphPad Software, United States). Data distribution was first assessed for normality using the Shapiro–Wilk test. For normally distributed data, comparisons between two groups were performed using unpaired two-tailed Student’s t-test, while comparisons among multiple groups were analyzed using one-way analysis of variance (ANOVA) followed by appropriate post hoc tests. For data that did not meet the assumption of normality, non-parametric tests were applied, including the Mann–Whitney U test for two-group comparisons and the Kruskal–Wallis test for multiple-group comparisons. For repeated-measures data (e.g., time-course measurements), two-way repeated-measures ANOVA was used, followed by post hoc Bonferroni’s multiple-comparison tests. Survival curves were analyzed using the Kaplan–Meier method and compared by the log-rank test. A value of *p* < 0.05 was considered statistically significant.

## 3 Results

### 3.1 Fabrication and physicochemical characterization of PNP-rtPA thrombolytic nanoparticles

To construct a biomimetic thrombolytic delivery platform with improved circulation stability and targeted fibrinolytic activity, we designed recombinant tissue-type plasminogen activator–functionalized platelet-membrane-coated PLGA nanoparticles (PNP-rtPA). As schematically illustrated in Figure 1A, perfluorohexane (PFH)–encapsulated PLGA nanoparticles were first synthesized via an emulsion–solvent evaporation method, followed by sequential coating with isolated platelet membranes and conjugation of DSPE-PEG-linked rtPA through amide coupling to yield the final rtPA-PMC-PNP formulation. Scanning-electron-microscopy (SEM) imaging revealed that the resulting nanoparticles exhibited uniform spherical morphology with smooth surfaces and a well-defined core–shell structure (Figure 1B). Dynamic-light-scattering (DLS) measurements showed a narrow hydrodynamic-diameter distribution centered around ∼180 nm, consistent with the nanoscale dimensions observed by SEM, and a slightly negative ζ-potential, reflecting successful membrane coating and surface modification (Figure 1C). These physicochemical features suggest good colloidal stability and potential for prolonged circulation. To further assess formulation reproducibility, we prepared three independent batches of PNP-rtPA and quantified both rtPA loading efficiency and retained enzymatic activity. As summarized in Supplementary Table S1, both parameters showed only limited inter-batch variation, indicating good batch-to-batch reproducibility of the formulation process.

To directly assess whether chemical conjugation and nanoparticle formulation affect the intrinsic enzymatic activity of rtPA, we performed a chromogenic substrate assay. As shown in Figures 1D,E, both the initial reaction velocity and substrate-cleavage kinetics indicate that DSPE-PEG-rtPA and PNP-rtPA retained a high level of enzymatic activity compared with free rtPA, indicating that the conjugation and formulation processes did not substantially impair rtPA function.

Western blot analysis further confirmed the preservation of key platelet-membrane proteins, including CXCR4, CD47, and ITGB3, in the hybrid membranes of PNP-rtPA (Figures 1F,G). Their expression remained stable under both physiological (pH 7.4) and thrombus-mimicking (pH 6.8) conditions, suggesting that key membrane protein expression and targeting-related properties were preserved after hybridization and surface conjugation. Consistently, incubation of PNP-rtPA in PBS supplemented with 1% BSA (pH 7.4, 37 °C) showed limited rtPA release under physiological conditions, supporting the stability of the surface-conjugated enzyme under simulated physiological conditions. To further evaluate the stability of surface-conjugated rtPA, we quantified rtPA release/detachment from PNP-rtPA under physiological (pH 7.4) and thrombus-mimicking (pH 6.8) conditions. As shown in Figure 1H, PNP-rtPA exhibited only limited rtPA release at pH 7.4 over 24 h, indicating good formulation stability under physiological conditions. In contrast, rtPA detachment was significantly increased at pH 6.8, suggesting that thrombus-like acidic conditions may facilitate localized rtPA release. These results support the notion that PNP-rtPA remains relatively stable in circulation while permitting enhanced rtPA availability in the thrombotic microenvironment. Importantly, although PNP-rtPA showed a slight reduction in clot-binding intensity compared with membrane-coated nanoparticles without PEG-rtPA modification, its thrombus association remained markedly higher than that of uncoated nanoparticles, indicating that PEG-based rtPA conjugation preserves most of the platelet membrane-mediated targeting capability (Figure 1I). Together, these results demonstrate the successful construction of rtPA-PMC-PNP nanoparticles with intact biomimetic surfaces and stable physicochemical properties suitable for targeted thrombolytic applications.

### 3.2 PNP-rtPA enhances thrombolytic efficacy in an electrical-stimulation-induced carotid thrombosis model

**FIGURE 2:**
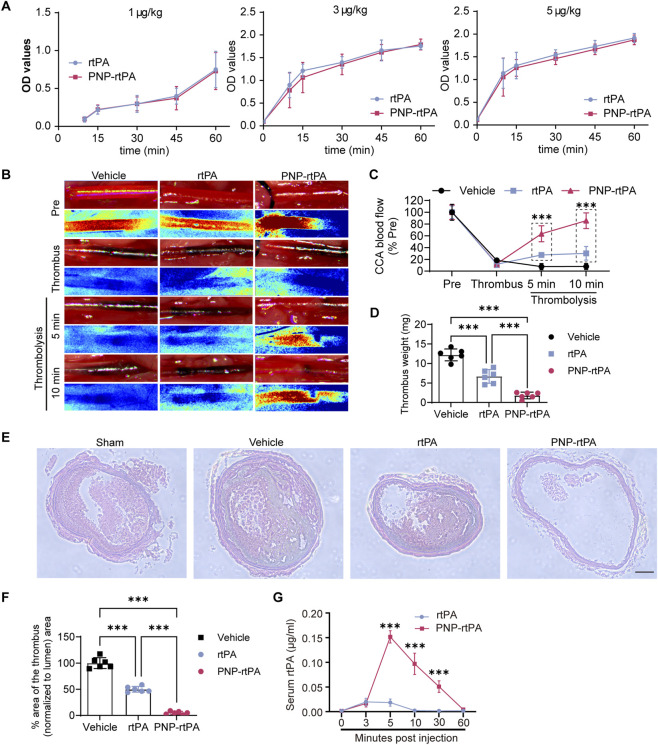
*In vitro* and *in vivo* thrombolytic efficacy of PNP-rtPA nanoparticles in a common carotid artery thrombosis model. **(A)** *In vitro* clot-lysis kinetics of PNP-rtPA and free recombinant tissue-type plasminogen activator (rtPA), showing comparable fibrinolytic activity between the two formulations. **(B,C)** Laser-speckle blood-flow imaging and representative photographs of thrombi in the common carotid artery (CCA) thromboembolism model at indicated time points after intravenous administration of vehicle, free rtPA, or PNP-rtPA. **(D)** Quantification of residual thrombus burden by weighing thrombosed CCA segments. **(E,F)** Hematoxylin-and-eosin **(H,E)** staining of carotid cross-sections and quantitative analysis of thrombus area following each treatment. Scale bar = 100 μm. **(G)** Plasma pharmacokinetics of human rtPA in mice after injection of free rtPA or PNP-rtPA, determined by ELISA. Data are presented as mean ± S.E.M. (n = 6 animals per group). Statistical analyses were performed using two-way repeated-measures ANOVA followed by Bonferroni’s multiple-comparison test for time-course data. *P < 0.05, **P < 0.01.

We next evaluated the thrombolytic performance of the PNP-rtPA both *in vitro* and *in vivo*. In a turbidimetric fibrin clot–lysis assay, PNP-rtPA displayed a comparable kinetic profile to that of free rtPA, confirming that the conjugation and encapsulation processes did not compromise the intrinsic enzymatic fibrinolytic activity of rtPA (Figure 2A). In this assay, whole blood was allowed to coagulate in 96-well plates, and subsequent clot dissolution by thrombolytic agents led to a time-dependent increase in supernatant absorbance, reflecting progressive fibrin degradation. The similar rise in optical density between PNP-rtPA and free rtPA groups indicated that rtPA retained high bioactivity after surface functionalization.

To assess thrombolytic efficacy *in vivo*, we established a low-frequency direct-current electrical stimulation (0.5 mA for 1.5 min)–induced common carotid artery (CCA) thrombosis model in mice. Real-time laser-speckle blood-flow imaging revealed that, compared with the vehicle group, PNP-rtPA treatment led to a rapid and sustained restoration of perfusion, whereas free rtPA (9 mg/kg) induced only a modest and transient increase in blood flow (Figure 2B). This limited effect of free rtPA is likely attributable to its short plasma half-life and the use of single-bolus intravenous injection rather than continuous infusion. Corresponding macroscopic images of excised carotid arteries corroborated these findings, showing markedly reduced residual thrombi in the PNP-rtPA–treated mice (Figure 2C). Quantitative analysis of thrombus burden by weighing thrombosed segments confirmed the superior fibrinolytic efficacy of PNP-rtPA compared with free rtPA and vehicle (Figure 2D). Histological analysis provided additional evidence for enhanced thrombus resolution. Hematoxylin-and-eosin (H&E) staining of carotid cross-sections demonstrated a substantially smaller intraluminal thrombus area following PNP-rtPA treatment than in the free-rtPA group (Figure 2E). Quantitative morphometric assessment indicated that PNP-rtPA achieved more complete recanalization and preservation of vascular wall structure, highlighting the improved thrombus-targeting and lytic efficiency conferred by platelet-membrane coating (Figure 2F).

To investigate the pharmacokinetic behavior underlying this enhanced efficacy, plasma rtPA concentrations were measured after systemic administration. As shown in Figure 2G, PNP-rtPA exhibited significantly prolonged circulation time and higher plasma retention of human rtPA compared with the free-rtPA formulation. The area under the concentration–time curve (AUC) was increased, suggesting reduced enzymatic degradation and enhanced stability *in vivo*. Collectively, these results demonstrate that the biomimetic PNP-rtPA nanoparticles preserve the fibrinolytic activity of native rtPA, achieve superior thrombus dissolution and vascular recanalization *in vivo*, and markedly extend systemic retention, thereby providing a promising thrombolytic platform with enhanced efficacy and pharmacokinetic advantage.

### 3.3 PNP-rtPA improves cerebral reperfusion and reduces infarct volume in a photothrombotic stroke model

**FIGURE 3:**
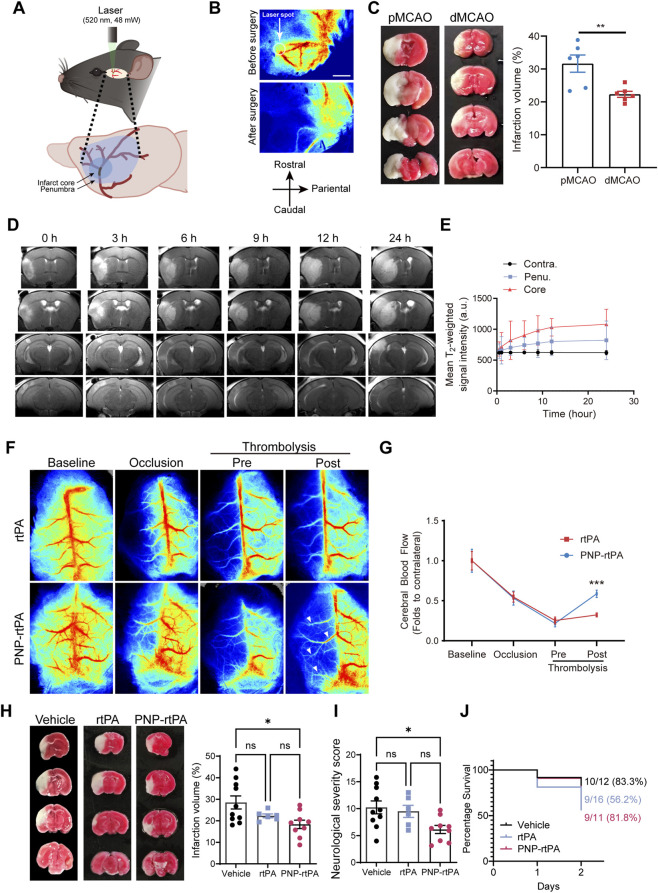
Therapeutic efficacy and extended thrombolytic time window of PNP-rtPA in a photothrombotic ischemic-stroke model. **(A)** Schematic illustration of the modified photothrombotic stroke model involving proximal middle cerebral artery occlusion (pMCAO). **(B)** Laser-speckle blood-flow imaging before and after occlusion showing successful induction of ischemia. **(C)** TTC staining of infarct regions in dMCAO and pMCAO occlusion models. **(D,E)** T_2_-weighted MRI scans and quantification of signal intensity in the ischemic core, penumbra, and contralateral hemisphere at 0, 3, 6, 9, 12, and 24 h after occlusion, demonstrating dynamic infarct evolution. **(F,G)** Laser-speckle perfusion maps before and after treatment with free rtPA or PNP-rtPA, showing improved reperfusion in the PNP-rtPA group. Regional cerebral blood flow was quantified as the ratio of ipsilateral (ischemic) to contralateral hemispheric perfusion at each indicated time point. **(H)** Representative TTC-stained coronal brain sections from vehicle-, free rtPA–, and PNP-rtPA–treated mice, and quantification of infarct volume based on TTC staining. **(I)** Neurological outcomes assessed by modified neurological severity score (mNSS) at 48 h post-treatment. **(J)** Survival curves of mice receiving vehicle, free rtPA, or PNP-rtPA during the 48-h observation period. Data are presented as mean ± S.E.M. (n = 11–16 animals per group). Statistical analyses were performed using an unpaired two-tailed Student’s t-test for panel C, two-way repeated-measures ANOVA for panels E and G, one-way ANOVA with Bonferroni’s post hoc test for panels H and I, and Kaplan–Meier analysis with log-rank test for panel J *P* < 0.05, P < 0.01, *P* < 0.001.

To further evaluate the therapeutic potential of PNP-rtPA in the cerebral circulation, we employed a modified photothrombotic ischemic-stroke model targeting the proximal middle cerebral artery (M3 segment) (Figure 3A). This approach used brief, high-intensity laser illumination to induce focal large-vessel thrombosis and produced a reproducible occlusion pattern representative of clinical large-vessel stroke. Laser-speckle perfusion mapping confirmed successful focal ischemia, as evidenced by an abrupt decline in regional cerebral blood flow at the laser-targeted site (Figure 3B). Comparative imaging of distal (dMCAO) and proximal (pMCAO) models by TTC staining verified that the pMCAO approach produced larger and more stable infarcts suitable for testing delayed thrombolysis (Figure 3C). To capture the dynamic evolution of cerebral injury, T_2_-weighted MRI was performed at multiple time points after occlusion. The ischemic core exhibited progressive hyperintensity, while the penumbral region showed transient signal elevation, indicating gradual infarct maturation over 24 h (Figures 3D,E). These results established a reproducible temporal profile for evaluating extended-window interventions.

Following intravenous administration of thrombolytics, PNP-rtPA treatment resulted in markedly improved reperfusion, as reflected by laser-speckle imaging showing a rapid and sustained recovery of cerebral blood flow (Figure 3F). In contrast, free rtPA induced only partial reperfusion within the same time frame. Quantitative analysis of ipsilateral-to-contralateral perfusion ratios confirmed that PNP-rtPA significantly enhanced regional cerebral blood flow compared with both vehicle and free-rtPA groups (Figure 3G).

The improved perfusion translated into robust neuroprotection. TTC-stained brain sections revealed a substantial reduction in infarct volume in PNP-rtPA–treated mice relative to the other groups (Figure 3H). Consistently, neurological-function assessment using the modified neurological severity score (mNSS) demonstrated significantly lower scores in the PNP-rtPA group, indicating better motor and sensory recovery (Figure 3I). Furthermore, survival analysis over a 48-h observation period showed a markedly higher survival rate among PNP-rtPA–treated mice compared with those receiving free rtPA (Figure 3J). Collectively, these findings demonstrate that PNP-rtPA confers enhanced thrombolytic efficacy and neuroprotection compared with conventional rtPA, leading to more effective cerebral reperfusion, reduced infarct burden, and improved short-term survival in ischemic stroke.

### 3.4 Prolonged circulation and enhanced brain accumulation of PNP-rtPA nanoparticles

**FIGURE 4:**
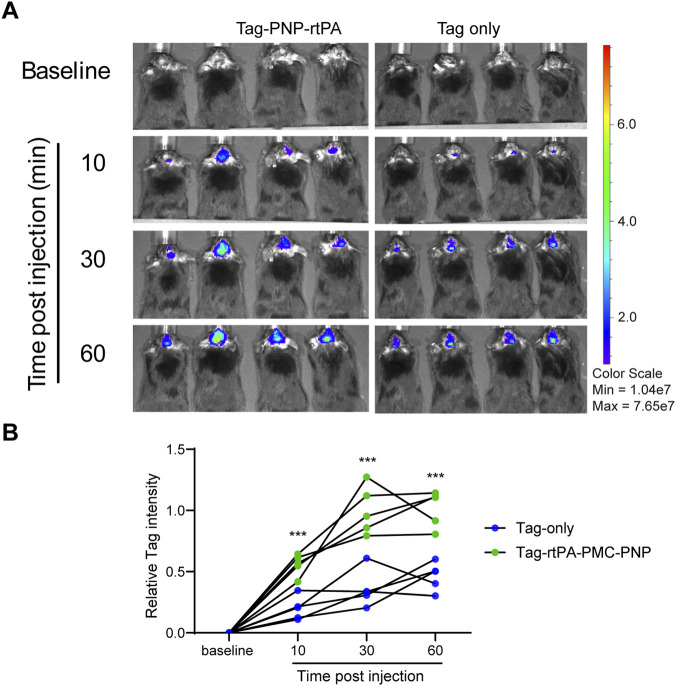
Pharmacokinetics and biodistribution of PNP-rtPA nanoparticles. **(A)** *In vivo* fluorescence imaging of mice intravenously injected with FITC-labeled PNP-rtPA or FITC-labeled PNP (control) at baseline and at 10, 30, and 60 min post-injection, showing real-time systemic distribution and clearance. **(B)** Quantification of fluorescence intensity in the brain area at each indicated time point, demonstrating prolonged circulation and enhanced brain accumulation of PNP-rtPA compared with control. Data are presented as mean ± S.E.M. (n = 6 animals per group). Two-group comparisons were analyzed by two-way ANOVA with Bonferroni’s post hoc test. ***P < 0.001.

To assess the *in vivo* behavior of the biomimetic thrombolytic nanoparticles, we performed real-time fluorescence imaging following intravenous injection of FITC-labeled PNP-rtPA or the tag-only control (uncoated PLGA nanoparticles lacking both rtPA and platelet membrane, with fluorescence labeling achieved via DSPE-PEG-FITC incorporation). Fluorescence signals were quantified within a predefined cranial ROI. Compared with the tag-only formulation, PNP-rtPA exhibited slower fluorescence decay and markedly prolonged vascular retention across the 60-min imaging period (Figure 4A). Quantitative analysis further showed that **c**ranial fluorescence intensity became significantly higher as early as 10 min post-injection (P < 0.001) and remained elevated throughout the observation window (Figure 4B).

Importantly, this fluorescence-based circulation profile is consistent with the extended systemic exposure observed in the pharmacokinetic assay (Figure 2G), in which PNP-rtPA maintained significantly higher plasma human rtPA levels over time compared with free rtPA. Together, these findings demonstrate that platelet-membrane-coated PNP-rtPA achieves prolonged *in vivo* retention and enhanced brain-associated accumulation, providing a pharmacokinetic foundation for its superior thrombolytic efficacy.

### 3.5 PNP-rtPA preserves blood–brain barrier integrity after thrombolytic therapy

**FIGURE 5:**
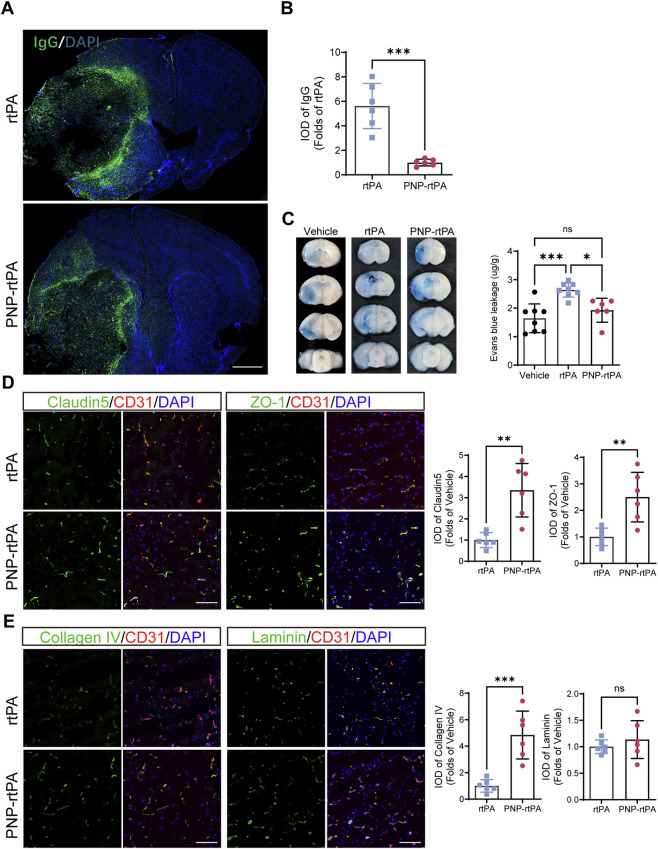
Assessment of blood–brain barrier integrity following thrombolytic treatment. **(A)** Representative immunofluorescence images showing endogenous IgG extravasation across the whole-brain sections after treatment with free rtPA or PNP-rtPA. Scale bar = 1000 μm. Quantification was performed within the peri-ischemic region adjacent to the infarct core to assess BBB permeability. **(B)** Quantification of IgG integrated optical density (IOD) in the peri-ischemic area between groups. **(C)** Evans Blue dye leakage assay and corresponding quantitative analysis of dye intensity in brain homogenates. **(D)** Immunofluorescence staining of tight-junction proteins claudin-5 and ZO-1. **(E)** Immunofluorescence staining of extracellular-matrix protein Collagen IV and Laminin. Scale bar = 100 μm. Relative IOD values were calculated as folds of the ischemic-side rtPA group. Data are presented as mean ± S.E.M. (n = 6 animals per group). Statistical analyses were performed using one-way ANOVA followed by Bonferroni’s post hoc test for multiple-group comparisons (panel C), and unpaired t-test for two-group comparisons (panels B, D, and E). *P < 0.05, **P < 0.01, ***P < 0.001.

Given the known risk of blood–brain barrier (BBB) disruption associated with thrombolytic therapy, we next evaluated BBB integrity after treatment with PNP-rtPA or free rtPA in ischemic mice. Immunofluorescence staining for endogenous IgG revealed extensive IgG extravasation in the peri-infarct regions of mice treated with free rtPA, whereas PNP-rtPA treatment markedly reduced IgG leakage (Figures 5A,B). Even under comparable infarct volumes, the extent of IgG extravasation was substantially lower in the PNP-rtPA group, indicating that the reduction in BBB permeability reflected a genuine protective effect rather than differences in lesion size. Consistent with these observations, Evans Blue dye leakage assays demonstrated a significant decrease in dye accumulation in brain tissue from PNP-rtPA–treated mice compared with those receiving free rtPA or vehicle (Figure 5C). These results collectively suggest that nanoparticle-mediated rtPA delivery mitigates BBB disruption typically induced by systemic thrombolysis.

To further examine the molecular architecture of the BBB, immunofluorescence analysis of tight-junction (TJ) proteins showed that PNP-rtPA preserved the continuous and organized distribution of Claudin-5 and ZO-1 along cerebral microvessels, whereas free rtPA caused marked discontinuity and fragmentation of these junctional proteins (Figure 5D). In addition, staining for extracellular-matrix (ECM) components revealed that Collagen IV expression was significantly better preserved in the PNP-rtPA group compared with free rtPA, while laminin showed a similar but non-significant trend toward preservation (Figure 5E). Together, these results indicate that PNP-rtPA maintains both endothelial junctional integrity and vascular basement-membrane structure, thereby preserving overall BBB architecture and function, consistent with the reduced vascular permeability observed in IgG and Evans Blue assays.

### 3.6 PNP-rtPA reduces hemorrhagic complications and neuroinflammation after ischemic stroke

**FIGURE 6:**
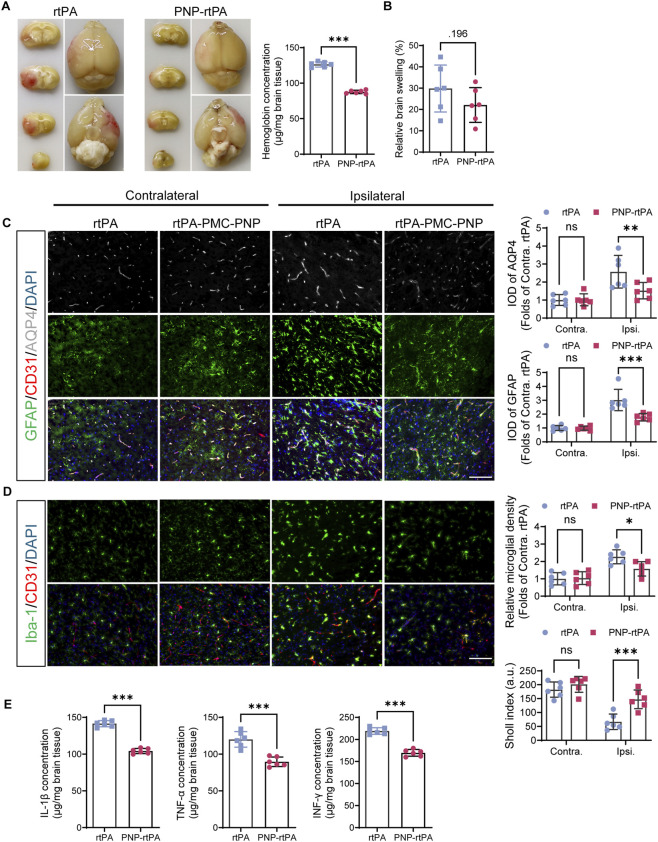
Safety evaluation of PNP-rtPA treatment. **(A)** Quantification of intracerebral hemorrhage by measuring hemoglobin content in brain homogenates 24 h after treatment with free rtPA or PNP-rtPA. **(B)** Measurement of brain-swelling ratio calculated from ipsilateral *versus* contralateral hemispheric volume. **(C)** Representative immunofluorescence staining for glial fibrillary acidic protein (GFAP), CD31, and aquaporin-4 (AQP4) showing astrocytic activation in the peri-ischemic region. Scale bar = 100 μm. **(D)** Representative immunofluorescence staining for ionized calcium-binding adapter molecule 1 (Iba-1) and CD31 illustrating microglial activation. Scale bar = 100 μm. Quantitative analysis of microglial density and Sholl index demonstrating that PNP-rtPA treatment reduced microglial proliferation while increasing morphological complexity, indicative of attenuated activation. **(E)** Serum inflammatory cytokine levels, including interleukin-1β (IL-1β), tumor necrosis factor-α (TNF-α), and interferon-γ (IFN-γ), determined by ELISA. Data are presented as mean ± S.E.M. (n = 6 animals per group). Statistical analyses were performed using one-way ANOVA for two-group comparisons within each region (contralateral or ipsilateral) across panels A–E. *P < 0.05, **P < 0.01, ***P < 0.001.

To evaluate the safety profile of PNP-rtPA in the context of thrombolytic therapy, we first quantified intracerebral hemorrhage using the hemoglobin assay. PNP-rtPA treatment resulted in significantly lower hemoglobin content in brain homogenates compared with free rtPA, indicating reduced risk of secondary hemorrhage (Figure 6A). Moreover, the brain-swelling ratio, calculated from ipsilateral *versus* contralateral hemispheric volume, was markedly decreased in the PNP-rtPA group (Figure 6B), suggesting mitigation of edema formation.

Immunofluorescence analysis further demonstrated reduced glial and vascular activation in PNP-rtPA–treated mice. Astrocytic markers GFAP and aquaporin-4 (AQP4) were less upregulated and more evenly distributed along microvessels, whereas CD31 staining indicated better preservation of vascular continuity (Figure 6C). Similarly, quantification of microglial morphology revealed a significant reduction in cell density and a concomitant increase in Sholl index within the peri-ischemic cortex following PNP-rtPA treatment (Figure 6D). These results indicate a reduction in microglial density accompanied by a shift toward a more ramified morphology, suggesting an overall attenuation of microglial activation.

In agreement with these histological findings, serum cytokine measurements showed significantly lower levels of pro-inflammatory mediators including interleukin-1β, tumor necrosis factor-α, and interferon-γ in the PNP-rtPA group compared with free rtPA (Figure 6E). Collectively, these results demonstrate that PNP-rtPA not only enhances thrombolytic efficacy but also reduces hemorrhagic complications, brain edema, and neuroinflammation, highlighting its favorable safety profile and therapeutic potential for ischemic stroke treatment.

## 4 Discussion

The present study introduces a biomimetic thrombolytic nanoplatform that regulates the bioactivity of rtPA at the neurovascular interface. From a biomaterials perspective, the major limitation of conventional rtPA therapy lies not only in its short circulation half-life (Yogendrakumar et al., 2024; Yaghi et al., 2017), but also in the lack of spatial control over fibrinolytic activity, which predisposes the cerebral vasculature to hemorrhagic injury. By integrating platelet-membrane-mediated biointerfacing with a polymeric nanocarrier, PNP-rtPA establishes a localized and vessel-compatible fibrinolytic microenvironment, thereby reconciling thrombolytic efficacy with vascular safety. In addition, chromogenic substrate assays confirmed that rtPA retained high enzymatic activity following conjugation and nanoparticle formulation, while release/detachment studies indicated minimal rtPA loss under physiological conditions but increased availability under thrombus-mimicking conditions. These results support the stability of PNP-rtPA in circulation and its capacity for localized thrombolytic activity.

In the broader context of biomimetic nanocarrier design, a variety of cell membrane-based strategies, including erythrocyte-, leukocyte-, and stem cell-derived systems, have been developed to enhance circulation time, immune evasion, and tissue targeting. Compared with these platforms, platelet membrane-based systems offer a unique advantage for thrombus targeting, as platelets inherently recognize vascular injury sites and fibrin-rich clots through adhesion-related mechanisms. Recent studies have highlighted the utility of platelet-mimetic nanoparticles for targeting injured vasculature, as well as the broader potential of biomimetic constructs, such as engineered microtissues, in cardiovascular repair and regeneration (Zhong et al., 2025). These findings underscore the versatility of biomimetic approaches across vascular diseases and support the use of platelet-derived membranes as a rational design for targeted thrombolysis in ischemic stroke.

Surface receptors such as integrin β3 and CXCR4 enable dynamic adhesion to fibrin-rich thrombi and activated endothelium, allowing the nanoplatform to exploit endogenous vascular recognition pathways rather than relying on synthetic targeting ligands (Chatterjee et al., 2015; Constantinescu-Bercu et al., 2022; Botero et al., 2020). Recent advances in platelet- and erythrocyte-derived nanocarriers further support their potential for precise thrombolytic and anti-inflammatory interventions (Li et al., 2023; Vincy et al., 2022; Zhao et al., 2025). In contrast to synthetic PEGylation or antibody modification, platelet membranes provide a dynamic and biocompatible interface that preserves native molecular interactions under physiological shear flow, thereby enabling material-guided targeting without introducing artificial ligands. These findings are consistent with the notion that PEG-based rtPA conjugation preserves most of the thrombus-targeting capability conferred by platelet membrane coating, although subtle effects on specific receptor-mediated interactions cannot be excluded.

Unlike free rtPA, which distributes systemically and indiscriminately activates downstream proteolytic cascades, the platelet-membrane-coated nanoplatform spatially confines fibrinolytic activity to the thrombus surface. Importantly, the preservation of tight junctions and basement membrane components observed after PNP-rtPA treatment reflects a material-mediated regulation of rtPA exposure at the vascular interface, rather than a direct pharmacological suppression of proteolytic pathways. This localized bioactivity minimizes excessive exposure of peri-ischemic microvessels to active rtPA, thereby reducing extracellular matrix degradation, tight-junction disruption, and subsequent hemorrhagic transformation.

The superior thrombolytic efficacy of PNP-rtPA observed in both the electrical-stimulation-induced carotid thrombosis and the modified proximal photothrombotic MCA occlusion models underscores the functional advantage of biomimetic delivery over conventional rtPA therapy (Figures 2, 3). Importantly, enhanced recanalization was achieved without compromising vascular structural integrity, highlighting a material-enabled dissociation between thrombolytic efficacy and vascular injury. In a reproducible large-vessel ischemia setting enabled by proximal M3 segment photothrombosis, the biomimetic nanoplatform achieved sustained reperfusion and efficient clot dissolution, demonstrating its ability to overcome the pharmacokinetic and spatial limitations of free rtPA. Whereas free rtPA, despite its potent fibrinolytic activity, often leads to transient or incomplete reperfusion due to rapid plasma clearance (half-life ≈5 min) and poor retention at the thrombus interface (Ta et al., 1989; Derhaschnig et al., 2024), PNP-rtPA enabled sustained perfusion recovery while preserving vascular wall structure. Such coordinated efficacy and safety likely arise from two synergistic material-enabled mechanisms: platelet-membrane-mediated anchoring that promotes site-specific accumulation at thrombotic sites, and nanocarrier-mediated protection that shields rtPA from premature degradation, thereby extending systemic bioavailability while restricting off-target vascular exposure. This mechanistic interpretation is supported by both the prolonged systemic rtPA exposure observed in pharmacokinetic analyses (Figure 2G) and the extended vascular retention revealed by *in vivo* fluorescence imaging (Figure 4).

Biomimetic nanocarriers provide a material-based strategy to enhance thrombus localization while mitigating systemic bleeding by prolonging rtPA circulation and promoting fibrin-specific interactions (Li et al., 2024; Guan and Dou, 2021). In this context, platelet membranes confer thrombus-targeting capability through their intrinsic repertoire of adhesion molecules, including glycoproteins that mediate interactions with activated endothelium and fibrin networks at sites of vascular injury (Qi et al., 2025). Consistent with this material–vascular interface, the superior perfusion recovery observed in the photothrombotic stroke model (Figures 3F,G) can be attributed not only to improved pharmacokinetic stability and targeted delivery, but also to a more physiologically compatible interaction between the biomimetic nanocarrier and the damaged vascular microenvironment.

Beyond enhancing thrombolysis, the biomimetic nanoplatform provides robust protection of the blood–brain barrier and an improved safety profile. The mechanism by which platelet-membrane coating mitigates rtPA-associated BBB disruption is likely multifactorial. First, surface localization of rtPA on the nanoparticle may reduce its nonspecific exposure to the endothelium, thereby limiting off-target proteolytic effects. In addition, platelet-derived membrane components may facilitate preferential adhesion to thrombotic sites, further restricting rtPA activity to the clot microenvironment. Although direct signaling effects of platelet membrane receptors cannot be excluded, the combined spatial confinement and targeting effects likely contribute to the observed preservation of BBB integrity.

The marked reduction of IgG and Evans Blue extravasation in PNP-rtPA–treated mice, even under comparable infarct volumes (Figures 5A–C), indicates that vascular preservation is independent of lesion size and instead reflects material-mediated confinement of rtPA bioactivity. By contrast, conventional rtPA can diffuse into peri-ischemic tissue and activate downstream proteolytic cascades, including PDGF-CC signaling and matrix metalloproteinases, leading to tight junction disruption and extracellular matrix degradation (Lewandowski et al., 2016; Su et al., 2008; Su et al., 2017; Kuo et al., 2020). By spatially restricting rtPA activity to the thrombus interface and enabling gradual release, the biomimetic nanocarrier minimizes off-target proteolysis, thereby reducing intracerebral hemorrhage and cerebral edema while maintaining efficient reperfusion (Figures 6A,B). Collectively, this shift from diffuse systemic fibrinolysis to localized, vessel-compatible thrombolysis reconciles the long-standing trade-off between efficacy and safety in rtPA-based therapy.

Consistent with the attenuation of cerebral edema, material-mediated vascular stabilization by PNP-rtPA was accompanied by reduced astrocytic swelling and reactive gliosis, as evidenced by decreased AQP4 and GFAP expression in the peri-ischemic cortex (Figure 6C). AQP4, the principal astrocytic water channel implicated in cytotoxic edema and BBB dysfunction after ischemia–reperfusion, showed a return toward baseline levels, indicating restoration of water homeostasis and alleviation of astroglial stress (Garcia Rios and Leon-Rojas, 2025). In parallel, microglial activation was markedly attenuated, as reflected by reduced cell density and simplified process architecture in Sholl analyses, consistent with a shift toward a quiescent surveillance phenotype rather than a pro-inflammatory state (Fukuda and Badaut, 2012). Importantly, these glial responses are unlikely to reflect a direct neuropharmacological or immunosuppressive action of rtPA but instead arise secondary to preserved vascular integrity and restricted plasma protein extravasation. Two converging material-enabled mechanisms may account for this effect: platelet-membrane camouflage that limits immune recognition and inflammatory signaling, and thrombus-confined rtPA release that minimizes BBB disruption and the subsequent infiltration of plasma-derived inflammatory mediators. Together, these processes dampen the acute neuroinflammatory cascade that typically amplifies tissue injury following reperfusion. By simultaneously stabilizing the vascular barrier, limiting glial reactivity, and restraining microglial overactivation, the biomimetic nanoplatform achieves an integrated form of neurovascular protection. This material-oriented strategy highlights the potential of bioactive thrombolytic interfaces to complement existing reperfusion approaches by improving safety without introducing additional neuropharmacological burden.

Finally, although the present study focuses on vascular and inflammatory outcomes, additional mechanisms such as oxidative stress and mitochondrial dysfunction may also contribute to reperfusion injury. Future studies incorporating redox-related analyses may further elucidate the protective mechanisms of PNP-rtPA (Hao et al., 2026). In addition, platelet membrane sourcing, standardization, and immunogenicity under repeated dosing will require systematic validation under Good Manufacturing Practice (GMP) conditions (Chugh et al., 2021; Liu et al., 2023).

Looking forward, integration of biomimetic thrombolytic nanoplatforms with image-guided or ultrasound-responsive systems could provide spatiotemporally controlled activation while further minimizing off-target proteolysis (Wang S. Y. et al., 2020; Wei et al., 2021). Moreover, coupling such material designs with artificial intelligence–assisted pharmacokinetic modeling and microfluidic vascular-on-chip validation may facilitate rational optimization of dosing strategies and safety profiles during preclinical development (Penaherrera-Pazmino et al., 2025; Liu et al., 2021; Ho et al., 2019). Collectively, PNP-rtPA exemplifies the convergence of biomaterials engineering and cerebrovascular medicine, illustrating how bioactive material interfaces can be leveraged to improve the therapeutic index of thrombolytic agents by balancing efficacy with vascular compatibility.

## Data Availability

The datasets presented in this article are not readily available because The datasets generated and analyzed during the current study are available from the corresponding authors on reasonable request. Requests to access the datasets should be directed to yz.ma@siat.ac.cn.
