# Supplementary material for: Platelet-membrane-coated nanoparticles enable safe and targeted thrombolysis with preserved neurovascular integrity
Source: Front Pharmacol. 2026 May 11;17:1825954. doi: 10.3389/fphar.2026.1825954 (PMC13199308; doi:10.3389/fphar.2026.1825954)
Supplement: Supplementary file 1 [file Table1.docx]

| **Batch** | **Initial rtPA input (mg)** | **Recovered/loaded rtPA (mg)** | **Loading efficiency (%)** | **Relative activity (%)** |
| --- | --- | --- | --- | --- |
| Batch 1 | 0.6 | 0.55 | 91.7 | 81.4 |
| Batch 2 | 0.6 | 0.57 | 95 | 84.8 |
| Batch 3 | 0.6 | 0.56 | 93.3 | 79.6 |
| Mean ± SD | — | — | 93.3 ± 1.7 | 81.9 ± 2.6 |

Supplementary Table S1

**Batch-to-batch reproducibility of PNP-rtPA preparation**

Three independent batches of PNP-rtPA were prepared using the same formulation protocol. rtPA loading efficiency was quantified by ELISA and expressed as the percentage of recovered rtPA relative to the initial input. Relative enzymatic activity was determined by chromogenic substrate assay and normalized to free rtPA. Data are presented as individual batch values and mean ± SD.
