# Supplementary material for: Platelet-membrane-coated nanoparticles enable safe and targeted thrombolysis with preserved neurovascular integrity
Source: Front Pharmacol. 2026 May 11;17:1825954. doi: 10.3389/fphar.2026.1825954 (PMC13199308; doi:10.3389/fphar.2026.1825954)
Supplement: Supplementary file 2 [file Table2.docx]

**Supplementary Table S2. Summary of animal usage across experiments**

| **Experiment** | **Figure** | **Group** | **Total animals (n)** | **Excluded** | **Deaths** |
| --- | --- | --- | --- | --- | --- |
| CCA thrombosis model | Fig. 2 | Vehicle | 6 | 0 | 0 |
|  |  | rtPA | 6 | 0 | 0 |
|  |  | PNP-rtPA | 6 | 0 | 0 |
| Photothrombotic stroke model (pMCAO) | Fig. 3B-D | PMCAO | 6 | 0 | 0 |
|  |  | dMCAO | 6 | 0 | 0 |
|  | Fig. 3F-J | Vehicle | 13 | 1 | 2 |
|  |  | rtPA | 16 | 0 | 7 |
|  |  | PNP-rtPA | 11 | 0 | 2 |
| BBB integrity analysis | Fig. 5 | Vehicle | 10 | 0 | 2 |
|  |  | rtPA | 10 | 0 | 2 |
|  |  | PNP-rtPA | 8 | 0 | 1 |
| Safety evaluation | Fig. 6A-B | rtPA | 13 | 1 | 6 |
|  |  | PNP-rtPA | 8 | 0 | 2 |
|  | Fig. 6C-E | rtPA | 12 | 0 | 6 |
|  |  | PNP-rtPA | 7 | 0 | 1 |
