# Supplementary figures and images for: Platelet-membrane-coated nanoparticles enable safe and targeted thrombolysis with preserved neurovascular integrity
Source: Front Pharmacol. 2026 May 11;17:1825954. doi: 10.3389/fphar.2026.1825954 (PMC13199308; doi:10.3389/fphar.2026.1825954)

Fig 1F

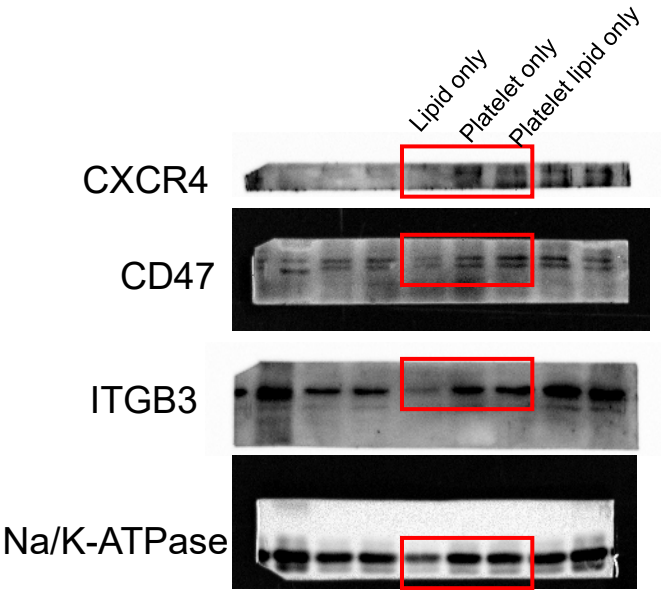

Fig 1G

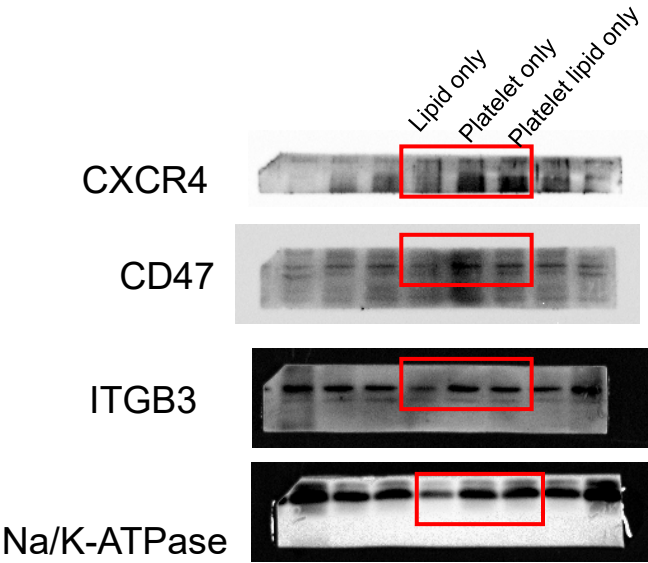

Supplement: Supplementary file 3 [file Image1.pdf]
